# Clinical commissioning and introduction of an in‐house artificial intelligence (AI) platform for automated head and neck intensity modulated radiation therapy (IMRT) treatment planning

**DOI:** 10.1002/acm2.14558

**Published:** 2024-11-06

**Authors:** Xinyi Li, Yang Sheng, Qingrong Jackie Wu, Yaorong Ge, David M. Brizel, Yvonne M. Mowery, Dongrong Yang, Fang‐Fang Yin, Qiuwen Wu

**Affiliations:** ^1^ Department of Radiation Oncology Duke University Medical Center Durham North Carolina United States; ^2^ Department of Information Systems University of North Carolina at Charlotte Charlotte North Carolina United States; ^3^ Department of Head and Neck Surgery and Communication Sciences Duke University Medical Center Durham North Carolina United States

**Keywords:** artificial intelligence, computer‐assisted radiotherapy, deep learning, head and neck cancers, radiotherapy planning

## Abstract

**Background and purpose:**

To describe the clinical commissioning of an in‐house artificial intelligence (AI) treatment planning platform for head‐and‐neck (HN) Intensity Modulated Radiation Therapy (IMRT).

**Materials and methods:**

The AI planning platform has three components: (1) a graphical user interface (GUI) is built within the framework of a commercial treatment planning system (TPS). The GUI allows AI models to run remotely on a designated workstation configured with GPU acceleration. (2) A template plan is automatically prepared involving both clinical and AI considerations, which include contour evaluation, isocenter placement, and beam/collimator jaw placement. (3) A well‐orchestrated suite of AI models predicts optimal fluence maps, which are imported into TPS for dose calculation followed by an optional automatic fine‐tuning. Six AI models provide flexible tradeoffs in parotid sparing and Planning Target Volume (PTV)‐organ‐at‐risk (OAR) preferences. Planners could examine the plan dose distribution and make further modifications as clinically needed. The performance of the AI plans was compared to the corresponding clinical plans.

**Results:**

The average plan generation time including manual operations was 10–15  min per case, with each AI model prediction taking ∼1 s. The six AI plans form a wide range of tradeoff choices between left and right parotids and between PTV and OARs compared with corresponding clinical plans, which correctly reflected their tradeoff designs.

**Conclusion:**

The in‐house AI IMRT treatment planning platform was developed and is available for clinical use at our institution. The process demonstrates outstanding performance and robustness of the AI platform and provides sufficient validation.

## 1 INTRODUCTION

Radiation treatment planning for head‐and‐neck (HN) cancer is challenging compared to other treatment sites. The entire planning process typically takes hours, including multiple rounds of communication between planners and physicians. Techniques have been developed to automate or partially automate the treatment planning process, such as kownledge‐based planning^1^, ^2^, ^3^, ^4^, ^5^, ^6^, ^7^, ^8^, ^9^ and mutlicriteria objectives.^10^, ^11^, ^12^, ^13^, ^14^, ^15^, ^16^ Recently, artificial intelligence (AI) has been rapidly evolving and has permeated many research fields and industries with breakthrough improvements in efficiency. As for treatment planning, previous studies have realized AI‐based or DL‐based auto‐planning for multiple sites incluing breast,^17^ pancreas,^18^ prostate,^19^, ^20^, ^21^ lung,^22^ and head‐and‐neck^23^ in research environments. An alternative approach is to predict dose distribution first and then generate plans via dose‐mimicking techniques.^24^, ^25^, ^26^, ^27^, ^28^ However, to be deployed in clinics, the AI or DL algorithms need a user‐friendly workflow that can be easily integrated into the clinical environment, as well as thorough commissioning and quality assurance (QA) similar to those performed for commercial treatment planning systems (TPS). AI‐based structure segmentation was one of the first AI techniques that had been implemented in clinics,^29^, ^30^ and AI‐based dose prediction and dose‐mimicking planning techniques were also implemented in clinics,^24^, ^31^, ^32^ while the majority of related studies were still in the research phase. One clinical implementation of AI in direct treatment plan generation was whole breast electronic tissue compensation (ECOMP) auto‐planning which has accumulated over 1000 patients.^33^, ^34^

Our previously developed AI planning algorithm employed DL to generate fluence maps from patient anatomy, bypassing the time‐consuming inverse planning. This AI algorithm was developed for intensity‐modulated radiation therapy (IMRT) plan generation for Head‐and‐Neck (HN) cases.^23^ The purpose of this study is to present a commissioning process of this AI system in the clinical environment so it can be safely and effectively used by treatment planners. Briefly, a graphical user interface (GUI) was developed between the AI algorithm and the commercial TPS (Varian Medical Systems, Milpitas, CA, USA) in the clinical environment. User operations are within the GUI embedded in the TPS, executing the core AI program remotely on a specifically configured server workstation. There are minimal human operations and changes to the standard clinical treatment planning workflow. In addition to the original AI model, five new AI models with different tradeoffs are provided, allowing planners to navigate the most suitable tradeoff. This AI platform was commissioned using 50 recent HN cases with various sub‐tumor sites. After sufficient validation and panel review, access to the platform has been granted to relavent dosimetrists and physicists in our institution since September 2022.

## 2 METHODS

### 2.1 Patient data and AI model training

Two hundred thirty one HN cases treated between 2013 and 2018 were included in the DL model development (200 training, 16 validation, and 15 testing). The primary planning target volumes (PTVs) for these cases were bilateral, mostly prescribed with 44 Gy (occasionally 50 Gy) in 2 Gy fractions. The boost targets were prescribed with 70 Gy (occasionally 60 Gy) in 2 Gy fractions and were treated in the sequential boost regime. This manuscript focuses on the primary plan generation, which usually takes the majority amount of the total planning time due to the larger size and more complicated geometry of the target compared with boost plans. A template‐based automatic planning for boost plan generation was included in the clinical GUI without any AI modeling.

All 231 cases were planned on TPS for six different tradeoff scenarios encompassing the most common decision choices in the clinical practice, as described in Table 1. These tradeoff scenarios were guided by experienced physicists and physicians, and the final plans reflected clinically viable tradeoffs and dose distributions.

**TABLE 1:** Plan labels and the corresponding tradeoff scenarios.

Plan label	Parotid sparing preference	PTV‐OAR^a^ preference
AI BP (Bilateral‐parotids and PTV)	Equally weighted left and right parotids	Prioritize PTV over OAR
AI BO (Bilateral‐parotids and OAR)	Equally weighted left and right parotids	Prioritize OAR over PTV
AI LP (Left‐parotid and PTV)	Left parotid only	Prioritize PTV over OAR
AI LO (Left‐parotid and OAR)	Left parotid only	Prioritize OAR over PTV
AI RP (Right‐parotid and PTV)	Right parotid only	Prioritize PTV over OAR
AI RO (Right‐parotid and OAR)	Right parotid only	Prioritize OAR over PTV

^a^ OAR: organ‐at‐risk.

For each case, planning CT images and structure contours were exported to generate the anatomical inputs for the AI models. Ten structures were modeled: primary PTV, primary clinical target volume (CTV), brainstem, cord+5  mm, left parotid, right parotid, oral cavity, larynx, pharynx, and mandible. Fluence maps of the ground truth plans were exported and padded into 128 × 128 pixels in 2.5 × 2.5 mm^2^ resolution, which were used for AI model training. Gantry angles are pre‐defined, starting from 180° and evenly distributed with 40° intervals for all plans. The 3D anatomical features of these structures were projected onto the Beam's Eye View (BEV) in the same dimension and resolution to match the fluence map, following these two projection principles: (1) intra‐structure projections truncate CT image values outside a specific structure mask; (2) interface projections truncate CT image values before the beamlet enters the body surface and after the beamlet exit on a specific structure's surface. Interface projections were generated for all 10 structures, and intra‐structure projections were generated for PTV and CTV. These 2D projections thus formed a 12‐channel anatomical input for the AI model. Details about the AI model structure and the model training is described in previous publication,^19^, ^23^ with a summary description attached as supplementary material A. Figures A1 and A2 illustrate the generator and discriminator networks’ architecture. To be deployed in clinic, the previously proposed model was separately trained using six sets of ground truth that corresponding to the six scenarios in Table 1.

To commission the AI platform, 50 more recent cases (2019–2022) were randomly collected with Institutional Review Board (IRB) approval. Among these cases, 38 were treated using 9‐beam IMRT, 10 using 11‐beam IMRT, and 2 with VMAT. These numbers reflect that the majority of HN cases’ primary clinical plans in our institution were 9‐beam IMRT. These cases included a variety of tumor types, where 35 were oropharynx, 6 were nasopharynx, 6 were larynx, and 3 were other types. These cases were collected to verify the AI platform's performance in the clinical environment compared with that for recent clinically treated plans.

### 2.2 AI planning workflow

The AI auto‐planning platform includes a Graphical User Interface (GUI) that seamlessly connects the AI auto‐planning elements and modules into the clinical treatment planning system (TPS). The GUI follows the current clinical planning workflow and guides the planner to review and inspect the AI outputs at each stage. Additionally, this GUI empowered clinical commission processes with high efficiency, where the commissioning cases could be planned, analyzed per commission criteria, and evaluated with clinical dosimetry metrics in the background automatically.

**FIGURE 1:**
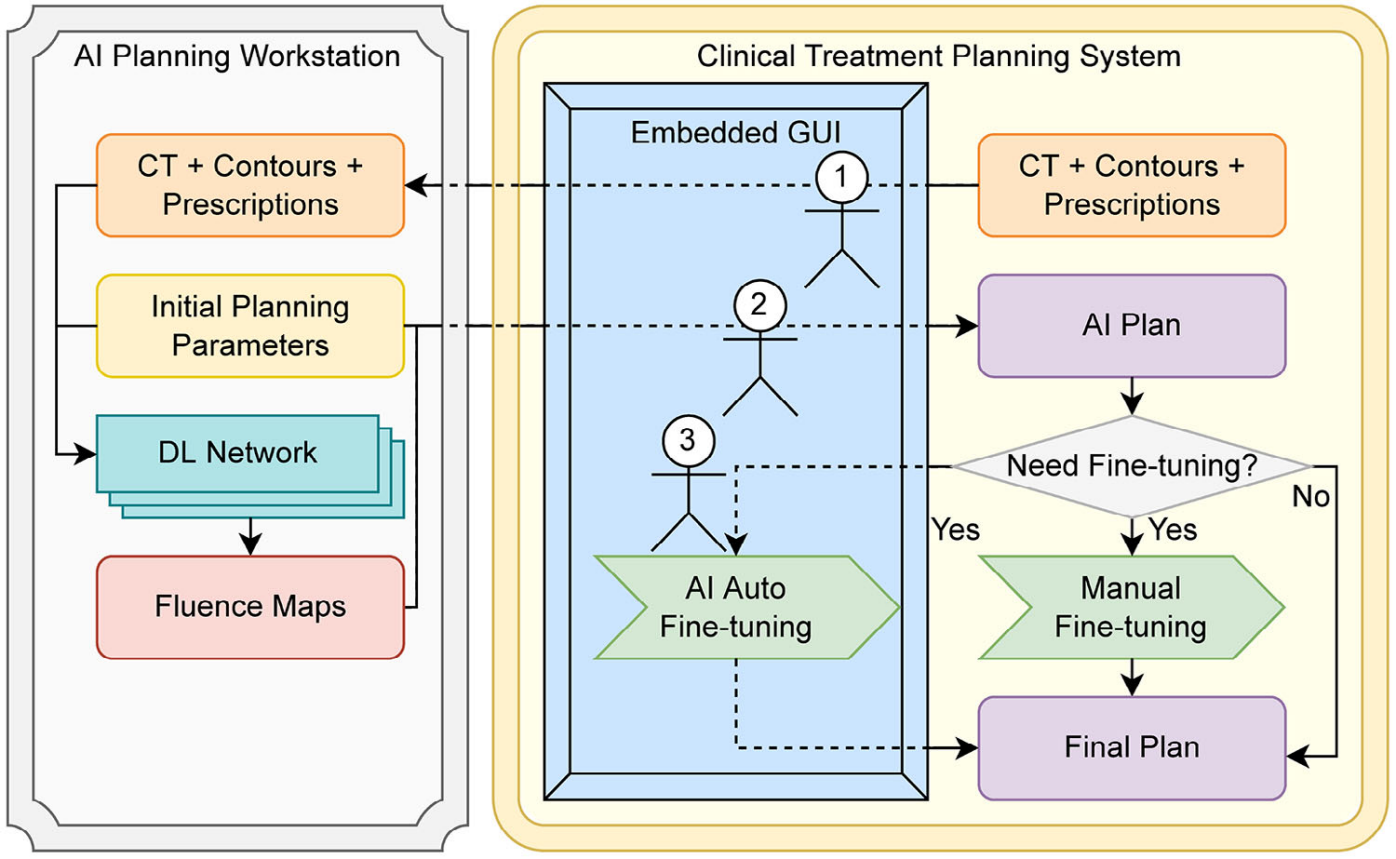
Treatment planning workflow with the AI platform.

As shown in Figure 1, the AI workflow is divided into three steps, represented by the three actor symbols in the figure that echo the planner's “pause‐review‐continue” process in the clinical workflow.

First, as indicated by the actor 1 symbol in Figure 1, after a planner inspects the CT image and structure contours, a GUI is presented within TPS's planning module. This GUI prompts the planner to confirm or select matching IDs of the structure set, course, plan, PTV, clinical target volume, and necessary OARs, similar to the “sanity checks” in manual IMRT/VMAT planning processes (Default IDs are automatically matched based on context). The planner needs to manually uncheck structures that are not supposed to be involved during optimization based on the physician's dose constraint sheet. After that, the AI platform automatically places the isocenter at the center point of the PTV with the highest prescription dose level, that is, boost PTVs when it applies. The isocenter will be changed to the center point of the primary PTV when the default isocenter position locates farther than 25 mm from the primary PTV's center point, otherwise the beam divergence can be too different from modeling conditions for the AI models based on our experience The isocenter shift is rounded to the nearest 5 mm for the convenience of patient setup. The AI platform also automatically sets a 9‐beam IMRT plan with optimal jaw positions and empty fluence maps. The AI platform places jaw positions using lungs and shoulders as anatomical landmarks. Each beam's collimator jaw size is defined individually mimicking the human planner's strategy of balancing beam entrance through shoulders and coverage of PTV volume. The beam gantry angle placements are evenly distributed with 40° intervals starting from 180 counterclockwise. Once completed, the planner is prompted to inspect the isocenter and jaw positions in TPS and make adjustments if necessary.

Second, as indicated by the actor 2 symbol in Figure 1, the AI platform remotely executes DL models on the server workstation, generating predicted fluence maps of each beam. The server workstation was specifically configured with the necessary GPU hardware and supporting software library for running these programs so that no extra configurations or hardware are needed on the clinical TPS workstations. The predicted fluence maps are imported into clinical TPS either automatically or manually, depending on the TPS versions/permissions.

Third, as indicated by the actor 3 symbol in Figure 1, the planner performs fluence map MLC leaf sequencing, dose calculation, and DVH calculation within the TPS (Eclipse AAA 15606, Smart LMC [15.6.06], and DVH Estimation Algorithm [15.6.06]) in the same way as manual IMRT planning. The dose distribution and DVH endpoints are carefully reviewed and evaluated by the planner, during which he/she makes the judgment on whether to perform an automatic fine‐tuning or whether the plan requires special modification at one or several local spots where manual fine‐tuning is warranted. For automatic fine‐tuning, the module is embedded in the AI platform and executes with one click. If planners decide to fine‐tune manually, a set of preset objectives will be added to the plan which are the same with those in auto fine‐tuning. All plans in the results sections were generated using these default objectives. Please refer to the supplementary materials for a video demo of how to use the AI platform and more technical details.

For clinical workstations, there is no extra requirements in software or hardware, either the TPS is locally installed or is a remote client. The AI planning workstation is required to be Windows system with stable internal internet access and a GPU. Currently, our AI workstation's GPU is Nvidia Quadro M4000 (8GB dedicated GPU memory). Software‐wise, the AI workstation requires MATLAB runtime 9.9 and Python 3.7.

### 2.3 AI planning platform commissioning

This AI platform was developed and commissioned to ensure its robustness and performance. The commissioning had three major steps. First, the AI platform went through an internal software review from an independent panel. The independent panel developed the institutional guidelines of software development. The panel reviewed the documentation of the AI Platform and evaluated the commission results thoroughly prior to approving it for clinical use.

The commission process was performed with 50 clinical HN cases that were not in the modeling process. The AI plan quality was reviewed jointly by experienced physicians and physicists. 3D dose distributions were visually examined, and critical dose–volume endpoints were statistically compared between AI plans and clinical plans as in Table 2. All plans were normalized as prescription dose (44 Gy) covering 95% of PTV (our institutional standard). The plans with 50 Gy prescriptions were scaled down to 44 Gy for statistical analysis. Target dose conformity was evaluated by conformity index (CI), which was defined as:

(1) $$CI=\frac{V_{PR}}{V_{PTV}},$$

where *V_PR_* is the prescription dose volume, and *V_PTV_* is the PTV volume. Target dose heterogeneity was evaluated by heterogeneity index (HI), which was defined as:

(2) $$HI=\frac{D_{2\%}-D_{98\%}}{R_{x}},$$

where *D*_2%_ is the dose to PTV's 2% and 98% volume, and *R_x_* is the prescription dose. Other dosimetric endpoints or metrics include BODY D_1cc_, brainstem and cord+5  mm D_0.1cc_, parotids, oral cavity, larynx, and pharynx's median dose, and total MU. The population median between clinical and AI plan pairs was compared using paired Wilcoxon signed‐rank tests with Bonferroni correction. The significant level was 0.05 by default.

**TABLE 2:** Statistics of median values (interquartile) of dosimetric endpoints of the 50 commissioning cases between clinical plans and AI plans.

	Bilateral parotids (18 cases)					
	Clinic	AI BP	AI BO	p1^b^	p2^c^	p3^d^
CI	1.20 (0.15)	1.09 (0.07)	1.17 (0.25)	0.0002^a^	0.0006^a^	0.2668
HI	9.3 (2.7)	9.6 (2.7)	15.3 (14.4)	0.0002^a^	0.5566	0.0002^a^
BODY D_1cc_ (Gy)	47.8 (1.2)	47.3 (1.0)	49.5 (3.8)	0.0002^a^	0.4997	0.0002^a^
Brainstem D_0.1cc_ (Gy)	15.7 (14.8)	14.9 (11.6)	14.1 (12.2)	0.4204	0.0429	0.0198
Cord+5 mm D_0.1cc_ (Gy)	31.0 (5.5)	29.2 (3.0)	26.0 (3.2)	0.0002^a^	0.0854	0.0012^a^
Parotid L D_median_ (Gy)	13.6 (4.6)	17.1 (4.9)	13.0 (3.6)	0.0002^a^	0.0279	0.0156
Parotid R D_median_ (Gy)	13.0 (4.1)	17.0 (3.6)	12.3 (3.8)	0.0002^a^	0.0025^a^	0.0123
Oral cavity D_median_ (Gy)	19.3 (12.8)	20.8 (11.7)	19.0 (8.1)	0.0057	0.2311	0.5862
Larynx D_median_ (Gy)	17.8 (8.7)	20.4 (5.3)	18.2 (5.2)	0.0833	0.8469	0.3591
Pharynx D_median_ (Gy)	34.1 (8.1)	35.4 (6.5)	31.9 (5.9)	0.0002^a^	0.8446	0.0475
Mandible D_1cc_ (Gy)	44.1 (5.7)	43.6 (8.6)	44.4 (4.9)	0.0033^a^	0.1701	0.6165
Total MU	1841 (483)	1473 (393)	1811 (410)	0.0002^a^	0.0057	0.4204

	Right parotid (16 cases)					
	Clinic	AI RP	AI RO	p1	p2	p3
CI	1.15 (0.06)	1.08 (0.03)	1.13 (0.08)	0.0004^a^	0.0011^a^	0.8767
HI	8.4 (3.7)	8.7 (2.0)	13.2 (4.4)	0.0004^a^	0.2553	0.0006^a^
BODY D_1cc_ (Gy)	47.3 (1.4)	47.6 (0.5)	49.3 (0.8)	0.0004^a^	0.1961	0.0005^a^
Brainstem D_0.1cc_ (Gy)	15.0 (4.1)	17.6 (7.1)	18.3 (8.8)	0.0494	0.0980	0.3520
Cord+5 mm D_0.1cc_ (Gy)	28.0 (6.4)	30.9 (3.2)	25.5 (2.1)	0.0004^a^	0.0879	0.1961
Parotid L D_median_ (Gy)	35.4 (11.2)	35.4 (7.1)	35.6 (7.5)	0.8361	0.3011	0.2775
Parotid R D_median_ (Gy)	11.5 (3.8)	10.7 (2.3)	9.7 (1.9)	0.0011^a^	0.1477	0.0072
Oral cavity D_median_ (Gy)	25.9 (13.4)	26.3 (8.7)	24.1 (6.3)	0.0004^a^	0.7960	0.0386
Larynx D_median_ (Gy)	18.0 (3.7)	19.9 (2.5)	17.3 (1.9)	0.0004^a^	0.0299	0.5014
Pharynx D_median_ (Gy)	34.2 (7.8)	34.2 (6.0)	30.8 (6.8)	0.0004^a^	0.9588	0.0045
Mandible D_1cc_ (Gy)	45.8 (1.5)	44.9 (0.4)	45.2 (0.7)	0.0052	0.0131	0.0980
Total MU	1994 (600)	1774 (281)	1849 (344)	0.0061	0.0787	0.2146

	Left parotid (16 cases)					
	Clinic	AI LP	AI LO	p1	p2	p3
CI	1.19 (0.09)	1.08 (0.05)	1.14 (0.09)	0.0004^a^	0.0004^a^	0.0113
HI	8.8 (2.8)	8.4 (0.7)	14.1 (4.5)	0.0004^a^	0.2553	0.0004^a^
BODY D_1cc_ (Gy)	47.6 (1.2)	47.4 (0.6)	49.7 (1.7)	0.0004^a^	0.1208	0.0004^a^
Brainstem D_0.1cc_ (Gy)	18.0 (6.6)	21.8 (4.1)	18.2 (2.0)	0.0013^a^	0.3011	0.4380
Cord+5 mm D_0.1cc_ (Gy)	32.1 (8.5)	30.9 (1.5)	27.1 (3.1)	0.0004^a^	0.8361	0.0262
Parotid L D_median_ (Gy)	12.8 (4.3)	11.1 (1.9)	9.9 (1.1)	0.0006^a^	0.0980	0.0113
Parotid R D_median_ (Gy)	29.1 (9.6)	34.4 (4.2)	34.0 (6.4)	0.3011	0.0113	0.0084
Oral Cavity D_median_ (Gy)	24.4 (6.4)	26.5 (5.9)	22.3 (6.4)	0.0004^a^	0.0299	0.4080
Larynx D_median_ (Gy)	16.9 (4.6)	21.0 (3.4)	18.2 (2.3)	0.0001^a^	0.0730	0.9341
Pharynx D_median_ (Gy)	33.2 (9.3)	33.8 (5.4)	28.3 (5.8)	0.0004^a^	0.2146	0.0084
Mandible D_1cc_ (Gy)	45.6 (1.6)	44.6 (1.0)	45.1 (1.0)	0.0200	0.0097	0.3011
Total MU	2030 (198)	1770 (260)	1874 (277)	0.0009^a^	0.0879	0.4691

^a^ Statistically significant in Wilcoxon signed‐rank tests with Bonferroni correction.

^b^ p1 was calculated between Clinical plans and AI BP/RP/LP plans.

^c^ p2 was calculated between Clinical plans and AI BO/RO/LO plans.

^d^ p3 was calculated between AI BP/RP/LP plans and AI BO/RO/LO plans.

Besides, 20 out of the 50 commissioning cases were randomly selected to evaluate the deliverability of AI plans. Each case randomly had one of the six AI plans examined via IMRT portal dosimetry QA (Varian Medical Systems, Palo Alto, CA, USA).

The AI platform needs to be QAed after any changes in the system settings or environments, including upgrades in Eclipse version, Linac machine modeling, computer OS upgrade, AI model training, etc., to ensure the AI predictions and plan settings are not changed.

## 3 RESULTS

Within current TPS settings (Eclipse version 15.6), the entire AI planning process on average takes about 10–15 min, including all manual operations and the three‐step pause‐and‐inspections described earlier. The actual fluence map prediction time for the entire 9‐beam set is less than 1 s per model with GPU (Nvidia Quadro M4000, 8GB dedicated GPU memory) on the dedicated workstation. Hence, the fluence map generation of all six models (or six tradeoff option plans) only takes 6 s.

**FIGURE 2:**
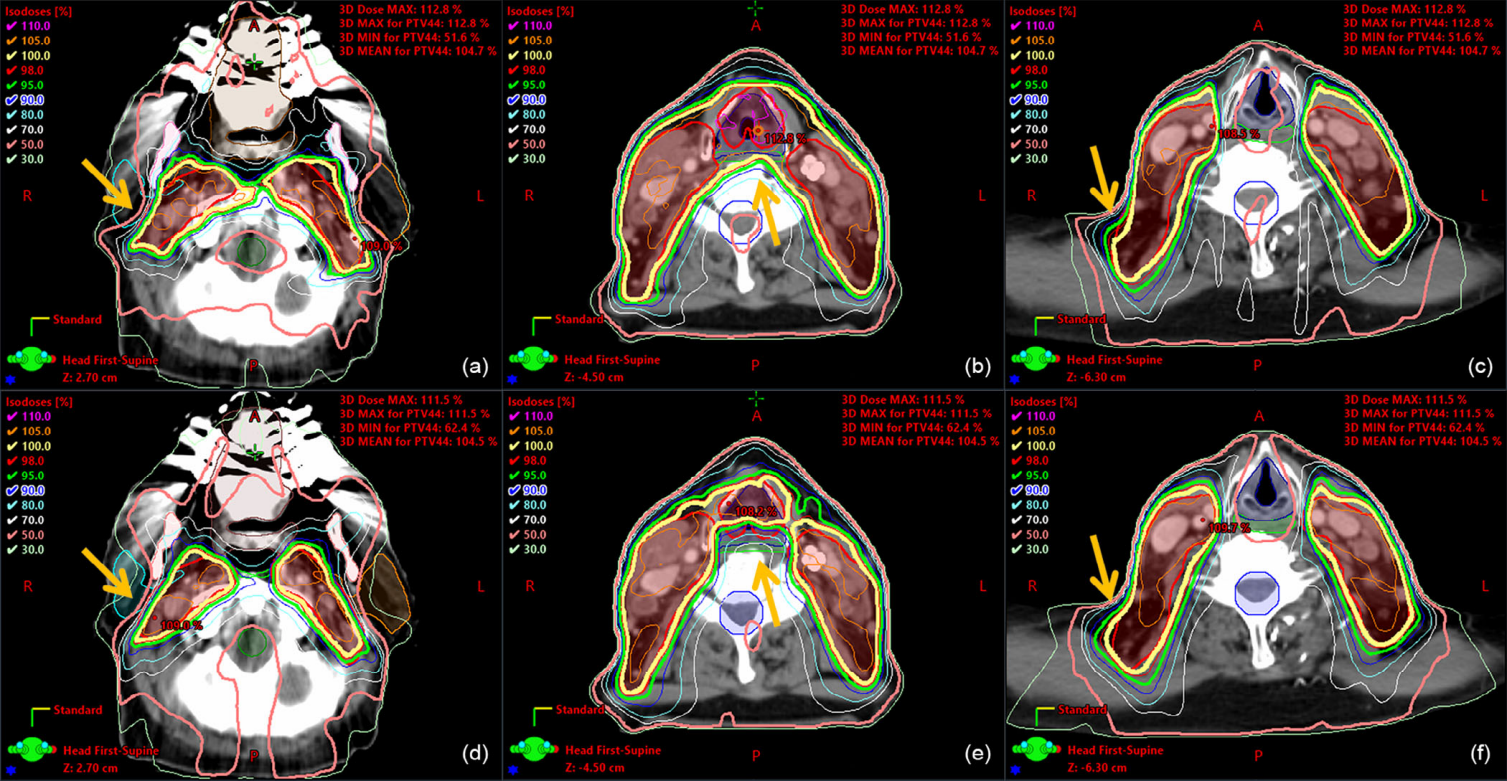
Dose distribution comparison of a commissioning case in axial view at different CT slices. (a–c) Clinical plan; (d, e) AI plan. Red: PTV44; Cyan: parotid right; Orange: parotid left; Green: brainstem; Blue: cord+5  mm; Dark blue: larynx; Green: pharynx; Brown: oral cavity. Yellow arrows indicate occasionally missed PTV coverage in both plans.

Figure 2 is an example of 3D dose distributions of a commissioning case, comparing the AI BP (Bilateral‐parotids and PTV) and the clinical plan. The BP plan offers equally weighted left and right parotids sparing and prioritizes PTV coverage over OAR sparing. In the AI BP plan, dose distribution showed satisfying conformity and dose fall‐off from the PTV. The PTV dose heterogeneity was acceptable with the hot spots located within the PTV. The coverage was acceptable with slightly missing near the edge of the PTV (shown by the yellow arrows). The OAR dose sparing was also clinically acceptable and comparable to the clinical plan. Overall, the AI plan and the clinical plan's dose distribution show highly similar characteristics. Of note, the clinical plan was an 11‐beam IMRT versus the AI plan was 9‐beam. Please refer to the supplementary materials for slice‐by‐slice 3D dose distribution comparisons.

**FIGURE 3:**
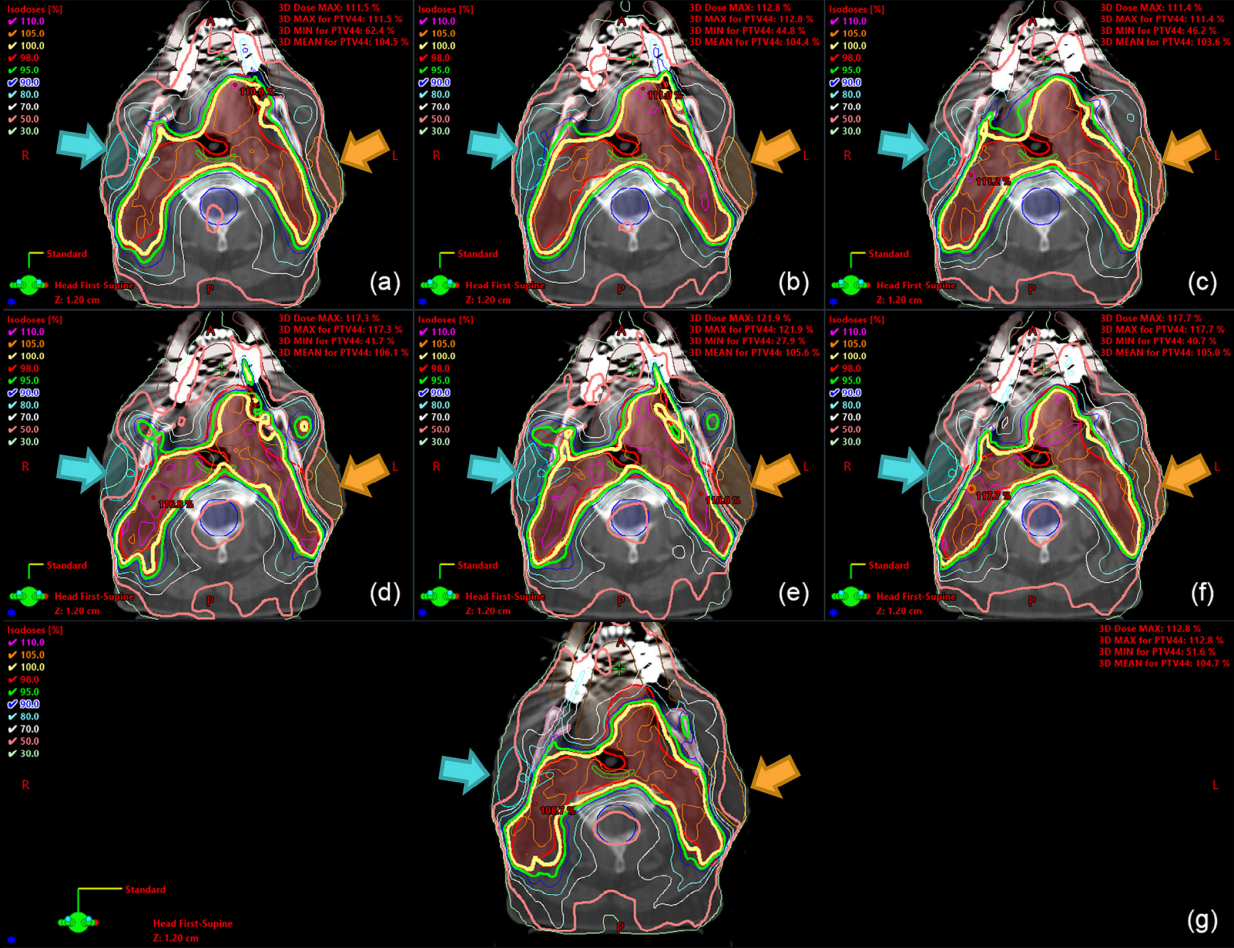
Isodose comparison between AI plans (a–f) and clinical plans (g). (a) BP, (b) LP, (c) RP, (d) BO, (e) LO, (f) RO. Red segment: PTV44; Green segment: brainstem; Cyan segment: right parotid; Orange segment: left parotid; Brown segment: oral cavity; Pink segment: mandible. Blue arrows indicate right parotid dose comparison, and orange arrows indicate left parotid dose comparison.

Figure 3 shows the isodose comparison between six AI plans with different tradeoffs ((a)–(f)) and the clinical plan (g) for the same commissioning case in Figure 2. Using the clinical plan's dose distribution as a reference, the different AI plans delivered tradeoff choices. Compared to the clinical plan, the right parotid in AI plans (c) RP, (d) BO, and (f) RO had better low‐dose sparing (30% isodose line in lime green, denoted by the cyan arrows); the same for the left parotid in AI plans (b) LP, (d) BO, and (e) LO (denoted by the orange arrows). However, AI plans (d) BO, (e) LO, and (f) RO had large hot spots over 110% in the PTV, indicating OAR sparing has reached its limit. During application, these hot spots could be removed by merging multiple AI plans or continuing inverse optimization.

Table 2 shows dosimetric endpoints for the clinical and AI plans. The 50 commissioning cases were divided into three groups based on the dosimetric tradeoff between parotids. In the first group, left and right parotids received similar median dose, and this group of clinical plans was compared with AI BP and AI BO plans. The second group's left parotids received lower median doses than right parotids and were compared with AI LP and AI LO plans; the third group's clinical plans were compared with AI RP and AI RO plans. Compared with the clinical plans, AI BP, LP, and RP plans have significantly better CI and are comparable for the other dosimetric endpoints except that AI BP plans have a higher right parotid median dose (p1). AI BO, LO, and RO plans have significantly higher HI and BODY D1cc, while other dosimetric endpoints are comparable with clinical plans, except that AI BP plans have lower cord+5  mm D0.1cc and pharynx median dose (p2). Compared with the corresponding AI *P, AI *O plans have significantly higher CI, HI, BODY D_1cc_, and most OAR dosimetric endpoints in AI *O plans are significantly lower (p3). Total MUs were comparable between clinical plans and AI plans. In summary, the AI plans correctly reflected tradeoffs between left and right parotids and between PTV and OAR as designed, while also achieving similar dosimetric endpoints compared with the clinical plans. Meanwhile, the AI *P and AI*O plans show reasonable divergence in dosimetric tradeoff options.

**FIGURE 4:**
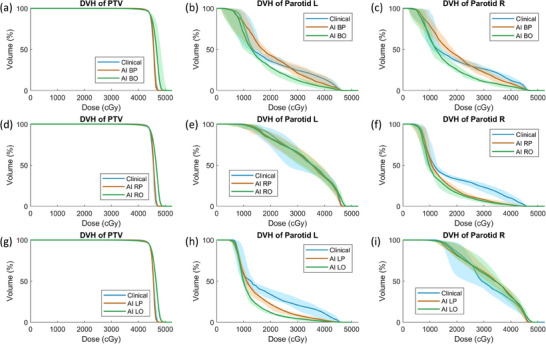
DVH comparison of the three groups of commissioning cases: (a)–(c) bilateral parotids (18 cases); (d)–(f) right parotid (16 cases); (g)–(i) left parotid (16 cases). The solid line stands for median DVH, and the colored region stands for 25–75 percentile.

Figure 4 shows DVH comparison of PTV, left parotid, and right parotid for the 50 commissionning cases in three groups. The median and percentiles were calculated over percentage volume for the same dose grid point. As shown in (a), (d), and (g), AI *O plans have higher PTV dose than clinical plans, whereas AI *P plans’ PTV DVH basically overlaps with those from clinical plans. For (a)–(c) bilateral parotids group, the clinical plans’ parotid DVH was between those from AI BP and AI BO plans in medium‐low dose region, and higher in high dose region. In (f), left parotid DVH for AI LP and AI LO was comparable to or lower than clinical plans’ DVH, similar for right parotid DVH in (h). Overall, the AI plans’ DVH behaves as we designed and shows promising plan quality.

IMRT QA using Portal Dosimetry (Varian Medical Systems, Palo Alto, CA, USA) was delivered for 20 of the 50 commissioning cases, including both AI plans (label shuffled) and 20 corresponding clinical plans (some were 11‐beam IMRT). The Γ passing rates (GPR)^35^ with criteria of 3%/2 mm for 180 AI plan fields (9 beams per plan) were 97.9 ± 2.5%, with three fields failing the test but passing with criteria of 2%/4 mm. Meanwhile, GPR for 188 clinical plan fields was 96.7 ± 4.1%, with 15 fields failing the test, but passed with criteria of 2%/4 mm. This result demonstrated the comparable deliverability of the AI plans to the clinical plans.

After the above validation of the AI platform's performance and robustness, the platform has been approved in the clinical TPS, and access has been granted to dosimetrists and physicists working on the HN site.

## 4 DISCUSSION

In the past decade, AI and DL algorithms have been developed and implemented in many medical fields. Due to their “black box” nature, sometimes researchers do not fully understand their mechanisms and thus do not know precisely when they make inaccurate predictions. This lack of understanding brings hesitation in the implementation of AI into clinical use.^36^ Therefore, although many reseach efforts have been put in DL‐based treatment planning, their clinical conversion is often detached afterwards. In this study, the AI platform was carefully designed to be integrated into the current clinic workflow. The AI auto planning was divided into several steps to allow planners to inspect outcomes at each step, and ensure they meet the planner's expectations and current planning guidelines and standards. Meanwhile, manual operations were reduced to the minimum, as most of the standard operations during the planning are automated with algorithms. The human planner only needs to focus on the outcome item that not meeting expectations or anatomical regions warranting manual or non‐standard intervention. Therefore, routine elements such as the contouring inspection, isocenter placement, and jaw setting for each beam, are automated with several clinic knowledge‐based algorithms. The fluence map prediction model applies DL algorithms that capture complex and high‐dimensional features in the anatomy and fluence map, allowing robust prediction and optimal dose distribution for daily clinical applications. The dose distributions and DVH are all calculated within the TPS, which means there will not be a lap between the predicted and the final plan quality. The AI planning platform also provides six plans with different tradeoff priorities simultaneously with no additional time cost, an enhancement to current planning workflow efficiency. With current manual planning, physicians, and planners often have to go through one or more iterations when special tradeoff consideration is necessary for a patient case. With AI planning, physicians are offered tradeoff decision support when these tradeoffs are presented together, similar to the Multi‐Criteria Optimization (Varian Medical Systems, Palo Alto, CA, USA).

The planning time, from the exporting contour structures to the final plan, is about 10–15 min, versus up to hours in the standard clinical routine.^37^ The platform is integrated with the current version of TPS (Eclipse v15.6), and the data communications between the AI algorithms and the TPS consume the bulk of AI auto‐planning time. The upgraded version of the TPS will drastically reduce the data communication time and hence the overall AI auto‐planning time. The platform has been available in clinical operation since September 2022. It is expected that the AI platform can significantly reduce planning time and improve plan quality consistency over time, particularly suitable for patients under clinical protocols and for planners who are new to HN cases.

One concern about the AI plan is its deliverability. Since it is integrated into the current clinical workflow, the AI‐generated fluence map is converted to the MLC leaf sequence by the clinical TPS, and the final dose distribution was computed based on these deliverable sequences, the same way as in the clinical planning routine. As expected, the final AI plans achieved the same deliverability as the manual IMRT plans, and our QA results also confirmed this observation.

Another limitation is that the number of OARs included is fixed in the AI plans. For some patients, submandibular glands, esophagus, brain, optic nerves, optic chiasm, lips, retinas, and lacrimal glands are possible OARs requiring additional sparing. When these extra OAR constraints are low priority, merging them with nearby modeled OARs could improve dose sparing for these un‐modeled OARs. However, if one of these un‐modeled OARs has a high priority for dose sparing, planners will need to manually adjust the AI plan via traditional inverse planning. If these un‐modeled OARs are contoured and prescribed with low dose constraints, it might indicate a relatively rare anatomical geometry of the patient. For example, optical structures are only contoured for patients with targets extending more superiorly than the majority of the patient cohort. This also indicates that the patient needs to be manually planned with additional caution. Training an AI model for an arbitrary number of OARs or arbitrary treatment sites is an exciting topic for our future study.

Finally, AI plans are currently limited to 9‐beam IMRT with evenly distributed gantry angles starting from 180°. These AI‐generated 9‐beam IMRT plans had comparable key dosimetric endpoints, regardless of the original clinical plan's beam configuration. Currently, the AI platform cannot be used to generate 11‐beam IMRT or VMAT plans, but it can be used as a reference or starting point for a more complex plan. Our group has also been investigating AI‐based VMAT planning,^21^, ^38^ which could be provided as a future option.

## 5 CONCLUSIONS

An AI platform for HN IMRT planning was developed and has been commissioned for clinical use. The AI platform is provided with simple operation instructions and clinically compatible workflow. In addition, it provides common tradeoff options between left and right parotids and between PTV and OAR. During the evaluation of the AI‐generated plans, the AI platform demonstrated robust performance and high efficiency. Future research will analyze data collected during the implementation phase of the AI platform and will aim to provide more functions to better serve the clinic.

## AUTHOR CONTRIBUTIONS

**Xinyi Li**: Methodology; software; data curation; investigation; writing—original draft. **Yang Sheng**: Conceptualization; methodology; software; writing—review & editing. **Q. Jackie Wu**: Conceptualization; methodology; writing—review & editing. **Yaorong Ge**: Conceptualization; methodology; writing—review & editing. **David M. Brizel**: Validation; writing—review & editing. **Yvonne M. Mowery**: Validation; writing—review & editing. **Dongrong Yang**: Software; data curation; validation; writing—review & editing. **Fang‐Fang Yin**: Validation; writing—review & editing. **Qiuwen Wu**: Conceptualization; methodology; writing—review & editing.

## CONFLICT OF INTEREST STATEMENT

The authors declare no conflicts of interest.

## Supporting information



SUPPORTING INFORMATION 1: Technical Details about AI Modeling

SUPPORTING INFORMATION 2: Video Demo of User Interface

SUPPORTING INFORMATION 3: Dose Distribution Comparison

SUPPORTING INFORMATION 4: Dose Distribution Comp

SUPPORTING INFORMATION 5: Dose Distribution Comparison
