# Supplementary material for: Clinical commissioning and introduction of an in‐house artificial intelligence (AI) platform for automated head and neck intensity modulated radiation therapy (IMRT) treatment planning
Source: J Appl Clin Med Phys. 2024 Nov 6;26(1):e14558. doi: 10.1002/acm2.14558 (PMC11712748; doi:10.1002/acm2.14558)
Supplement: Supplementary file 1 — SUPPORTING INFORMATION 1: Technical Details about AI Modeling [file ACM2-26-e14558-s004.docx]

**Supplementary material A. AI model training**


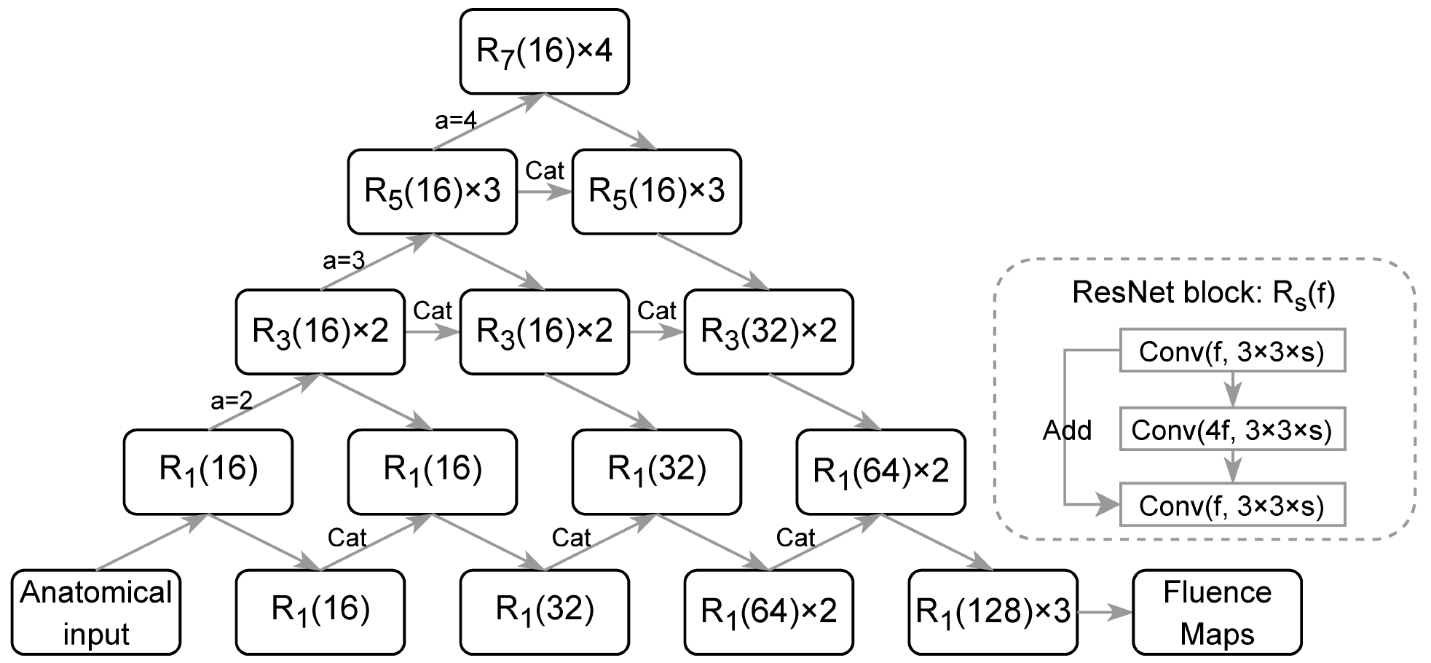


Figure A1. The generator’s network structures in AI model training. The insert shows the structure of a ResNet block, which consists of 3 convolutional layers and an add layer. Abbreviations: f: number of filters; s: filter size in the third dimension; a: Atrous rate; Cat: concatenation layer; Conv: convolutional layer.


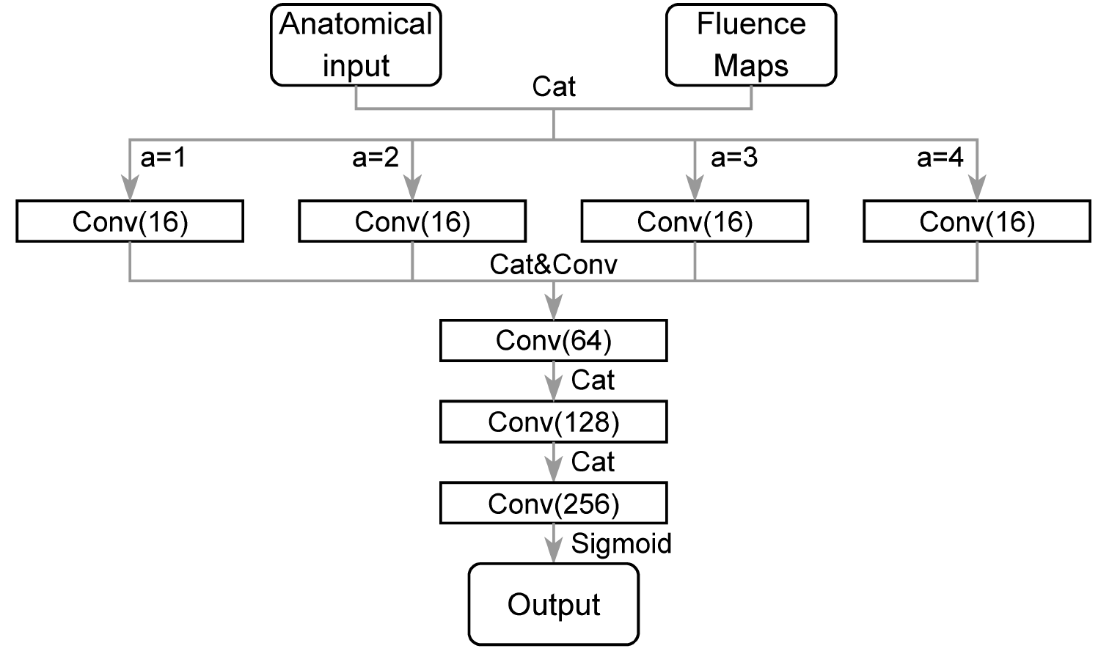


Figure A2. The discriminator network structure in AI model training. Abbreviations are the same as in Figure A1.

Figures A1 and A2 showed the DL network structures in the AI planning platform, also described previously in the feasibility study^1^. The networks were trained under conditional Generative Adversarial Network (pix2pix) architecture^2^, where a generator and a discriminator were simultaneously trained against each other. The generator in Figure A1 was PyraNet, where 28 ResNet^3^ blocks were concatenated into the shape of a pyramid. Each ResNet block has 3 convolution layers and the first layer’s output was added to the third layer’s output, as shown in the upper inset. After the last ResNet block, the filter number was reduced to 1 and thus generates fluence maps. The discriminator in Figure A2 was a customized DenseNet^4^. Except for fluence maps, the anatomical input was also fed to the discriminator as the “condition”. “Cat&Conv” indicates that the previous layer’s output was concatenated with the current layer’s output. All filter sizes were 3×3×1. After the last convolution layer, a sigmoid activation layer normalized the output values to between 0 and 1.

The discriminator’s loss function was Mean Squared Error (MSE). It was trained by an Adam optimizer (learning rate = 2×10^-4^). The generator’s loss function was Haar wavelet loss, which was calculated by summing the Mean Absolute Error (MAE) of the prediction error and its level 1 to 6 Harr wavelet transform results. The generator was combined with the discriminator, and the discriminator’s parameters are fixed during the training of the combined model. This combined model was trained by an Adam optimizer (learning rate = 2×10^-5^). The combined model’s loss was:

$$\begin{aligned} {loss}_{combined}={loss}_{generator}+{loss}_{discriminator}\times200 \#(1) \end{aligned}$$

This model was separately trained for different ground truth fluence maps with different tradeoffs. Training of each model takes about 800 epochs, which takes around 100 hours on a workstation with an Intel® Xeon® W-2195 CPU (2.3 GHz, 256 GB RAM in total) and 4 NVIDIA Quadro RTX 8000 GPUs (48 GB dedicated GPU memory each, used in parallel). The final model was the epoch with the smallest validation loss. Although the training process may take weeks, each model’s prediction time is around 1s on the designated workstation with one GPU (Nvidia Quadro M4000, 8GB dedicated GPU memory).

1. Li X, Wang C, Sheng Y, et al. An Artificial Intelligence-Driven Agent for Real-Time Head-and-Neck IMRT Plan Generation using Conditional Generative Adversarial Network (cGAN). *Med Phys*. Feb 12 2021;doi:10.1002/mp.14770

2. Isola P, Zhu J-Y, Zhou T, Efros AA. Image-to-Image Translation with Conditional Adversarial Networks. *arXiv e-prints*. 2016:arXiv:1611.07004. Accessed November 01, 2016. <https://ui.adsabs.harvard.edu/abs/2016arXiv161107004I>

3. He K, Zhang X, Ren S, Sun J. Deep Residual Learning for Image Recognition. *arXiv e-prints*. 2015:arXiv: 1512.03385.

4. Huang G, Liu Z, van der Maaten L, Weinberger KQ. Densely Connected Convolutional Networks. *arXiv preprint arXiv:160806993*. 2016;
